# A giant right atrial myxoma with pulmonary arterial hypertension

**DOI:** 10.11604/pamj.2015.21.96.7055

**Published:** 2015-06-05

**Authors:** Ihsen Zairi, Khadija Mzoughi, Zouhayer Jnifene, Sana Fennira, Fathia Ben Moussa, Sofiene Kammoun, Mohamed Mounir Dhiab, Sondos Kraiem

**Affiliations:** 1 Department of Cardiology, Habib Thameur Public Hospital, Bab El Fallah, Tunis, Tunisia; 2 Departement of Cardio Vascular Surgery, La Rabta Hospital, Tunis, Tunisia

**Keywords:** Atrial myxoma, surgery, cardiac tumors

## Abstract

Here we report a case of a right atrial mass that morphology mimicking myxoma, in a young patient with no past medical history. The mass was pathologically confirmed to be symptomatic and surgical removal was successfully done.

## Introduction

Atrial myxomas are the most common primary heart tumors. Because of nonspecific symptoms, early diagnosis may be a challenge. The majority of myxomas are located in the left atrium and have variable clinical presentation. We report a case of a large myxoma in the right atrium, which is an uncommon location for this type of tumor.

## Patient and observation

**Figure 1:**
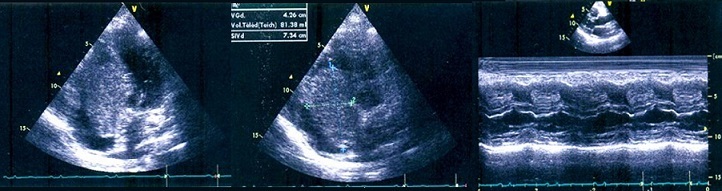
Transthoracic echocardiogram: the four chambers and parasternal TM view showing the hyperechoic mass in the right atrium which was wide based and apeended to the interatrial septum and prolapsing through the tricuspid valve

**Figure 2:**
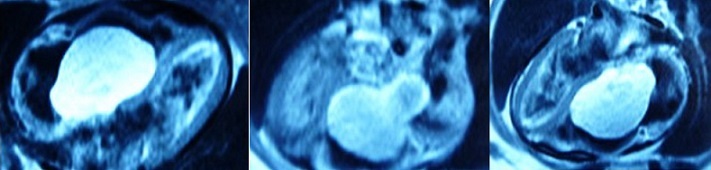
Magnetic resonance imaging: showing the mass in the right atrium

**Figure 3:**
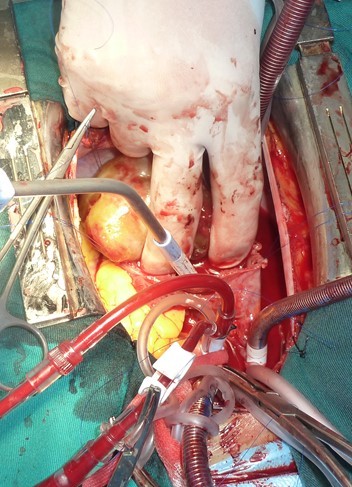
Intraoperative photograph of the tumor projecting from the right atrium and attached to the interatrial septum

**Figure 4:**
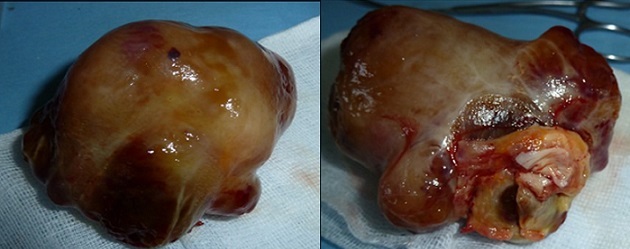
Explanted tumor, a lobulated mass

A 44-year-old woman was admitted to Habib Thameur Hospital because of progressive dyspnea and asthenia. No past medical history of cardiovascular risk factors. Clinical examination showed awell-looking woman with a facial pigmentation. No signs of congestion was noticed. Cardiac auscultation revealed normal heart sounds and a systolic murmurin Tricuspid Valve Area. Trans thoracic echocardiogram exam demonstrated amobile right atrial mass enclosed in the right ventricle and mesures 7 cm of diameter (Figure 1). There was an important pulmonary arterial hypertension. Magnetic Resonance Imaging reveals an intracardiac tumor between right atrium and ventricle (Figure 2). The diagnosis of right atrial myxoma was made and the tumor resection surgery was indicated. Median sternotomy was performed. A dilated right atrium was opened after cardio pulmonary by pass was initiated. When the right atrial was dissected, it was found a cardiac mass in the right atrium measuring 7 cm of maximal diameter, appended to the interatrial septum that looks like a typical cardiac myxoma (Figure 3, Figure 4), which is enclosing and occupying almost the whole right ventricle. The tumor was resected and then the residual septal defect was repaired with a Dacronpatch. A tricuspid annuloplasty was done because the tricuspid annulus was dilated. After the operation, the patient was transferred to the intensive care unit and was extubated. Trans thoracic echocardiogram exam reveals regression of pulmonary arterial hypertension. The postoperative course was uneventful and he was dischargedsix days later. This patient was viewed three months later and she was totally asymptomatic.

## Discussion

Primary cardiac neoplasms are rare and occur with an estimated incidence of0.0017% to 0.19%, representing less than 5% of all heart tumors [1]. Myxoma is the most prevalent primary heart tumor and are the most common benign tumours of the heart [2]. Cardiac myxomas usually develop in the atria. About 75% of myxomas arise in the left atrium. Right atrial myxomas are rare as myxomas are estimated to occur in the right atrium in only 15-20% of the cases [3]. Only 3 to 4 percent of myxomas are detected in the left ventricle, and only 3 to 4 percent in the right [4]. Myxomas occur in all age groups but are particularly frequent between the third and sixth decades of life [5]. Clinical presentation is varied and may mimic arterial embolism or symptoms of cardiac, infectious, malignant and immune diseases, therefore making their diagnosis challenging [2]. RA myxomas may remain asymptomatic [6] or eventually cause constitutional signs and symptoms, including fever, weight loss, arthralgias, Raynaud phenome non, anemia, hyper gamma globulinemia, and anincreased erythrocyte sedimentation rate due to the production of interleukin-6 [7]. These symptoms disappear after the tumoris removed. In this report, our patient denied arthralgias, and anemia but complained offacial pigmentation as the only constitutional sign. However, the most common manifestation is dyspnea (in 80% of patients), and right heart failure has been reported. Dyspnea on exertion wasfound inthis case [7]. Echocardiography, including the transesophageal approach, is the most important means of diagnosis (sensitivity of up to 100%); CT and MRI may also be helpful. Resonance imaging and CT provide information regarding tissue characteristics and allow an excellent over view of cardiac and paracardiac morphology. MRI, in this case, showed an enlarged cardiac silhouette with an expansive ovoid mass in the RA with a density greater than that in the heart muscle [8]. The treatment is Complete resection of the tumor an dits implantation [9].

## Conclusion

The RA should always be considered in the differential diagnosis of a right-sided heart mass, especially when the patient shows signs and symptoms of heart failure with uncertain etiology.
